# Barriers and facilitators to smoking cessation in a cancer context: A qualitative study of patient, family and professional views

**DOI:** 10.1186/s12885-017-3344-z

**Published:** 2017-05-19

**Authors:** Mary Wells, Patricia Aitchison, Fiona Harris, Gozde Ozakinci, Andrew Radley, Linda Bauld, Vikki Entwistle, Alastair Munro, Sally Haw, Bill Culbard, Brian Williams

**Affiliations:** 1 NMAHP Research Unit, University of Stirling, Scion House, Stirling, FK9 4HN UK; 2 School of Medicine, University of St Andrews, St Andrews, KY16 9TF UK; 3 NHS Tayside, Public Health Directorate, Kings Cross Hospital, Dundee, DD3 8EA UK; 4 Insitute of Social Marketing, Faculty of Health Sciences and Sport, University of Stirling, Stirling, FK9 4LA UK; 5 Health Services Research Unit, University of Aberdeen, Aberdeen, AB25 2ZD UK; 6 Faculty of Health Sciences and Sport, University of Stirling, Stirling, FK9 4LA UK; 7 School of Health and Social Care, Edinburgh Napier University, 9 Sighthill Court, Edinburgh, EH11 4BN UK

**Keywords:** Smoking cessation, Patients, Health professionals, Family members, Cancer, Qualitative research

## Abstract

**Background:**

Continued smoking after cancer adversely affects quality of life and survival, but one fifth of cancer survivors still smoke. Despite its demands, cancer presents an opportunity for positive behaviour change. Smoking often occurs in social groups, therefore interventions which target families *and* individuals may be more successful. This qualitative study explored patients, family members and health professionals’ views and experiences of smoking and smoking cessation after cancer, in order to inform future interventions.

**Methods:**

In-depth qualitative interviews (*n* = 67) with 29 patients, 14 family members and 24 health professionals. Data were analysed using the ‘Framework’ method.

**Results:**

Few patients and family members had used National Health Service (NHS) smoking cessation services and more than half still smoked. Most recalled little ‘smoking-related’ discussion with clinicians but were receptive to talking openly. Clinicians revealed several barriers to discussion. Participants’ continued smoking was explained by the stress of diagnosis; desire to maintain personal control; and lack of connection between smoking, cancer and health.

**Conclusions:**

A range of barriers to smoking cessation exist for patients and family members. These are insufficiently assessed and considered by clinicians. Interventions must be more effectively integrated into routine practice.

## Background

Cancer survival is significantly worse in smokers [1, 2], and stopping smoking after cancer diagnosis improves survival in a number of tumour types [3]. A systematic review of the influence of smoking cessation on prognosis after early stage lung cancer diagnosis found that five-year survival rates in 65 year old patients were estimated to be 33% in continuing smokers and 70% in those who stopped [4]. Continued smoking after diagnosis produces a range of adverse outcomes [5], including greater treatment toxicity [6] and reduced quality of life [7].

Despite the fact that people who do stop smoking after a cancer diagnosis can derive clear physical and psychological benefits [5], surveys suggest that around 20% overall continue to smoke [8–10], and that this is more likely in younger people, sexual minority groups [11] and those without a partner [12]. A recent study from the United States (US) suggests that one tenth are still smoking 9 years later [13]. Few data exist on how many survivors access smoking cessation services although we know that the vast majority of smokers in general populations want to stop in any given year, although the proportion varies between countries [14]. In addition, people who do use cessation services are more likely to stop and remain abstinent [15].

There is an extensive literature on the role of healthcare workers in providing smoking cessation interventions, illustrating the importance of infrastructure and managerial support [16], education and feedback directed at how to talk about smoking and how to implement evidence-based smoking interventions [17–20], as well as efficient referral and monitoring systems [16]. However, there is still a significant lack of research into smoking cessation interventions in the cancer field [5, 21].

The period around a cancer diagnosis presents an opportunity for behaviour change in patients [22] and family members [23]. Recent studies have found higher ‘quit rates’ in smokers *with* cancer compared to smokers in the general population [24], indicating that they may be more receptive to cessation support. Indeed, the potential reach and uptake of smoking cessation services may be increased in smokers who have cancer [25]. However, cancer diagnosis is emotionally demanding for most people and consequently, smoking cessation intervention strategies must be sensitive to the multiple problems faced by patients and their families. Interventions also need to take account of the beliefs, prescribing behaviours and approach of health professionals towards smoking cessation in cancer patients, which may be less than optimal [26–28]. International surveys of cancer clinicians confirm that only a minority routinely offer or refer patients to smoking cessation support [29, 30]. Few qualitative studies have explored either the reasons for this or how patients feel about smoking cessation in the context of a cancer diagnosis [31]. To our knowledge, there are no studies of the views and experiences of family members who smoke.

Cancer has a significant impact on patients’ friends and family members, and psychological interventions oriented to the family unit can have beneficial effects on a range of caregiver outcomes [32]. Smoking (and non-smoking) are often part of the identity of a social group and so can act either as a barrier or facilitator to smoking interventions [33], and studies show that family members of people with cancer are more likely to be motivated to quit [34] and more likely to access smoking cessation services [35]. However, despite clear evidence that family members’ beliefs and behaviour influence smoking cessation [33, 36, 37], current primary prevention interventions focus almost exclusively on individuals, and there has been very little research conducted on the particular issues facing patients and family members who smoke, after a recent cancer diagnosis.

In order to inform interventions that are sensitive to the cancer context and likely to be effective at reducing smoking in practice, this study explored the experiences and views of patients, family members and health care professionals towards smoking and smoking cessation around the time of cancer diagnosis.

## Methods

We conducted a qualitative study in Scotland using in-depth interviews, in order to develop the theoretical and empirical basis of an intervention to improve uptake of existing effective smoking cessation services: an approach consistent with the United Kingdom’s Medical Research Council (MRC) complex interventions framework [38, 39]. National Health Service (NHS) management and ethics approval were granted (13/ES/0032–22/5/13 and 6/6/13). Our research questions were: -

1. What are cancer patients’ and family members’ experiences of engaging with current NHS smoking cessation services, and which forms and constituent characteristics of such services were found helpful or unhelpful?
2. What do patients and family members who have not previously engaged with smoking services believe are the key barriers and potential facilitators to encouraging successful uptake of current forms of smoking cessation services in the first 6 months after a cancer diagnosis?
3. What do health professionals believe are the key barriers and facilitators to discussing smoking cessation with patients and family members in the first 6 months after a cancer diagnosis?
4. What are health professionals’ views regarding the organisational, psychosocial, ethical and clinical factors that may affect delivery, uptake and engagement with smoking cessation services, among patients with cancer and their family members?
5. What are the key characteristics and dimensions of a context-sensitive intervention that would render it acceptable, feasible and effective in increasing uptake of smoking cessation services (from the perspectives of patients, family members and health professionals)?

### Recruitment and sampling strategy

Three samples were recruited: patients, family members and health professionals. Inclusion criteria for our patient sample were: adults over 18 years of age who could speak English; more than 2 weeks but less than 3 years from diagnosis of lung, head & neck, colorectal or cervical cancer; on active follow up; and currently smoke or smoked until diagnosis. We excluded patients who were judged by the clinical team to be too distressed to participate.

One member of the team (PA) screened hospital clinic records to identify eligible patients, who were then approached at out-patient clinics. Patients willing to participate completed a screening questionnaire to assess: current smoking, smoking dependency, intention to stop smoking, use of cessation services and family structure (see Table 1). Recruitment aimed to achieve sampling diversity in relation to age, gender, diagnosis, treatment intent, socio-economic status (as indicated by Scottish Index of Multiple Deprivation (SIMD) [40]), time since diagnosis and current smoking status. Recruitment (and interviews) took place over a 16 month period and sampling diversity was achieved by continuing to screen for eligible patients firstly by cancer type (by attending different cancer outpatient clinics and screening case notes for eligible patients) and then inviting all eligible patients to participate until target numbers were reached. The socioeconomic diversity of the sample reflected the patient demographic of clinic attenders.

**Table 1:** Screening questionnaire used in recruitment and sampling

Are you a current smoker or a recent ex-smoker (i.e. since diagnosis)?
Smoker/Recent ex-smoker
How many cigarettes per day did you smoke over the 6 months prior to diagnosis and how soon after waking did you smoke?
Do you have at least one close family member who is either a current smoker or who stopped smoking after your diagnosis? Y/N
Does your family member live with or apart from you? W/A
Have you or your family member had any previous experience of using smoking cessation services? Patient Y/N Family member Y/N
Are you or your family members currently considering smoking cessation?
Patient Y/N Family member Y/N
Stage Diagnosis stage/Treatment stage/Follow-up
Period since diagnosis:
Age

Family members or partners/close friends of cancer patients were eligible to take part if they were smokers/recent ex-smokers. Due to difficulties in achieving target numbers in this group, recruitment used a range of strategies: patients nominated one or two close relatives or friends who were smokers or recent ex-smokers at the time of interview; family members were given information about the study directly by a researcher or nurse; advertising the study in a local newspaper; and advertising the study within smoking cessation groups (see Table 2). Only family members (rather than close friends of patients) took part in interviews.

**Table 2:** Characteristics of study participants (Patients and Family Members)

	Patients (*n* = 29)	Family Members (*n* = 14)
Gender:		
Female	13	8
Male	16	6
Age:		
30–50 yrs	5	4
51–60 yrs	7	3
61–70 yrs	12	5
71–80 yrs	5	1
81–90 yrs	0	1
Place of recruitment:		
Via oncology clinic	29	9
Via newspaper advertisement	N/A	3
Via NHS Smoking Cessation Group	N/A	2
Relationship of family member to patient participant:		
Spouse/partner	N/A	5
Daughter		1
Parents		2
Sister		1
Recruited independently of patient		5
Scottish Index of Multiple Deprivation (SIMD): 2012 Quintile:		
1 (Most deprived)	7	8
2	6	3
3	5	1
4	9	1
5 (Least deprived)	2	1
Smoking status (at time of interview):		
Smoker	15	11
Ex-smoker (since around diagnosis)	14	3
Cancer type:		
Head & Neck	10	N/A
Colorectal	7	
Lung	7	
Gynaecological	5	
Time since diagnosis:		
≤ 6 months	13	N/A
7–12 months	5	
13–18 months	8	
> 18 months	3	
Treatment intent:		
Radical	23	N/A
Palliative	6	

Drawing on research team clinical expertise, a purposive sample of health professionals involved in cancer and smoking cessation services were identified in order to seek the views of a range of professionals who might inform the study. This included oncologists, clinical nurse specialists, therapy radiographers, pharmacists, clinic and ward nurses, General Practitioners (GPs) and smoking cessation advisors.

Invitation letters and information sheets were distributed at clinics, sent via email to professionals or posted to those responding to the newspaper advertisement. Interested professionals, patients or family members were later telephoned to arrange an interview. Informed consent was sought immediately before interview.

Interview sample: Sixty seven interviews were conducted across the three participant groups:

- Sample 1: Twenty-nine patients with cancer who were current smokers or recent ex-smokers (Table 2), from 58 who were approached. Joint interviews were held with four patients/family members.
- Sample 2: Fourteen family members who were current smokers or recent ex-smokers (Table 2).
- Sample 3: Twenty-four health professionals from oncology, primary care and smoking cessation services (Table 3). All those approached agreed to be interviewed.

**Table 3:** Characteristics of study participants (Health Professionals)

	Health Professionals (*n* = 24)
Professional role:	
Clinical Nurse Specialist	5
Medical Specialist (Consultant Oncologist/Surgeon/Specialist Registrar)	5
General Practitioner	2
Senior Nurses (Consultant/Advanced Practitioner/Team Leader/Senior Nurse)	6
Therapy Radiographers	2
Pharmacy (Oncology & community)	2
Oncology Support Worker	1
Member of NHS Smoking Cessation Team	1
Work type:	
Acute hospital	19
Community hospital or setting	5
Gender:	
Female	17
Male	7

### Data collection

Patient, family member and professional interviews were conducted concurrently to allow emergent themes to be explored between the groups in an iterative fashion. Topic guides incorporated Leventhal’s ‘commonsense’ model [41] and advice from our patient advisor in the research team (Table 4). Leventhal’s model proposes that a person’s mental model of an illness has an impact on behaviours in response to that illness, and comprises: his or her sense of ‘illness identity’ (illness diagnosis and associated symptoms); causes; timeline (is the illness acute, chronic, or cyclical?); consequences (e.g., social, financial); and control (the degree to which the patient feels he or she has control over the illness) [42]. In relation to smoking behaviour within the cancer context, we sought to examine the nature of the patients’ and family members’ understanding of the link between cancer diagnosis and prognosis and smoking behaviour. Interviews with patients and family members explored their previous and current smoking behaviour and beliefs, the place of smoking within family and wider social networks and experiences of talking about cancer and smoking following a personal or family member’s cancer diagnosis. Interviews with health professionals explored current practices, experiences, concerns, opportunities and barriers to smoking cessation. The acceptability, feasibility and potential features of smoking cessation interventions for patients and family members were also explored.

**Table 4:** Interview Topic Guide

Main topic areas	
1. Context – participant’s experience and understanding of diagnosis, care, treatment	
2. Smoking behaviour and beliefs – smoking history, feelings and beliefs about smoking, relationship with health	
3. Smoking and social networks – views and behaviours of others	
4. Attempts at smoking cessation – how these felt, use of services, experiences	
5. Accessing healthcare as a smoker – discussions about smoking/cessation	
6. Experiences of cancer and smoking – discussions, information, support, connections made, changes in behaviour or feelings, use of services, attitudes towards smoking now, family support, difficulties and challenges	
7. Views about smoking advice intervention for people with cancer and families – what would work, not work, challenges, benefits	
8. Feelings about the interview, talking about smoking	

All interviews were conducted by an experienced qualitative researcher (PA), digitally-recorded and transcribed verbatim. Most patient and family member interviews took place in their own homes. Health professionals were interviewed at a workplace location. Interviews lasted between half an hour and 90 min. All participants were reassured that interviews were confidential and that any reported data would be anonymised.

### Data analysis

Data were analysed using the constant-comparative technique within the ‘Framework’ method [43]. Interview transcripts were managed using NVIVO (v10). Three members of the research team were involved in reviewing transcripts and enabling identification of emergent themes for subsequent exploration. An analysis Working Group (MW, PA, FH, GO) designed interim coding frameworks, which Steering Group members helped develop further after use on a selection of finalised transcripts. PA and FH then applied the analytical framework to transcripts and captured subsequent, emergent themes. Matrices and charts were used to compare themes within and across participant groups and to identify key analytical themes and supporting quotations. Attention was paid to exploring a variation of views and seeking explanations for disconfirming cases.

## Results

Fifteen out of 29 patient participants and 11 out of 14 family members were current smokers at the time of interview. The others all reported having stopped smoking at or around the time of diagnosis. Qualitative interviews with patients and family members revealed several barriers to smoking cessation and provided insights into their experiences of and perceptions towards smoking cessation services. Few participants had used UK (NHS) smoking cessation services and some held negative views as to their appropriateness. In addition, few participants recalled meaningful discussions with health professionals about smoking. Interviews with health professionals revealed concerns about sensitivity, perceptions of responsibility for talking about smoking, awareness of services and views of potential facilitators to smoking cessation.

Three key themes emerged to explain patients’ and family members’ continued smoking, and we present these as *barriers to smoking cessation*. These include: the stress experienced following a diagnosis; a desire to maintain personal control and a sense of ‘normal’ self; and lack of belief in or acceptance of the connection between smoking, cancer and health. We illustrate each theme with quotes from both patients and family members, where appropriate. Quotes are coded as follows: P (patient) or F (family member) – Study number – Type of cancer (patients only) – Smoker or non-smoker. Information on gender has been removed.

### Stress experienced following diagnosis

The period following a diagnosis of cancer was experienced as particularly stressful for patients and family members. For some patients and family members, smoking was used as a way of helping to cope with their stress.

*‘[Stopping smoking is] very much in my mind at the moment but I'm very unable to stop at the moment because I'm a bit uptight about the chemotherapy and family are here all the time and, while I feel well, I feel quite stressed a lot of the time….I'm quite positive about the treatment, but I do feel the need for a cigarette sometimes.’ (P28-Colorectal-Smoker)*

*‘But I think [after diagnosis] at that time it was because I wasn’t working…I had nothing else to do but think about like “What’s this going to affect, what effect is it going to have on the family?” and I think that built up a stress which led me to smoking more.’ (P54-Gynae-Smoker)*

*‘I can go one day without or two days without, I can still stop smoking, but it's just when everything piles up it's like a […] comfort, that's what it's like.’ (P47-Head&Neck-Smoker)*

*…what's happened to [wife who has received cancer diagnosis]] in the past six weeks, she’s went from a normal life to what she’s got now and the smoking helps me just to get over it…because I can go outside and sit on my own and think about it…’ (F6-Smoker)*

The degree to which smoking was perceived as a coping mechanism was highlighted by two patients, both of whom identified a strong addictive element to their smoking. For these patients, even thoughts of stopping smoking aroused a stress response which they anticipated they would have difficulty dealing with.

*‘…I just couldn’t get over that first hurdle not smoking, if they [cigarettes] weren’t there I’d panic, honestly I would.’* (P29-Lung-Smoker)

*‘[…] I hate myself for smoking. I have got to stop. Now the question is stopping in a way that causes me, but more importantly my wife, the least distress because…when I stop I can… be nasty, like I say, I’d pick a fight with the sofa.*’ (P10-Lung-Smoker)

### A desire to maintain personal control

The desire to exercise personal control and choice over smoking behaviour *within the context of* the cancer diagnosis emerged as an underlying barrier to cessation for patients. Following diagnosis, patients who experienced ‘nagging’ or pressure by relatives to stop smoking resisted and resented this, emphasising that a decision to stop smoking was theirs alone. There was a sense in which patients wanted to assert themselves and, for some, the decision to continue smoking was a way of doing this.

*‘I think a little bit of it is so many people saying “Stop”, that your mind is saying, “No, I’ll stop when I want to”. I'm not having people telling me to stop.’ (P31-Head&Neck-Smoker)*

*‘But I’ll have my cousins and that saying “You shouldn’t be smoking anyway”…and I just go “Yes, whatever”. But, no, each to their own really.’ (P54-Gynae-Smoker)*

*‘I'm damned if I'm going to be told what to do by them [family members].’ (P8-Colorectal-Smoker)*

In contrast to the experience of some patients, the 11 family members who continued to smoke did not recall experiencing significant pressure to stop smoking either from their relative who had cancer or from other family members.

### Lack of ‘coherence’ between smoking, cancer and health

Patients who continued to smoke tended to express limited perceptions of and uncertainty about the risks and consequences of smoking and, in turn, the benefits of stopping. Where they made links between smoking and health in their interviews, they expressed these primarily in terms of smoking as a cause (or not) of cancer rather than in relation to recovery or future health. In some instances, patients explained and justified continued smoking by drawing on messages received from healthcare professionals which, they perceived, minimised the connection between smoking and cancer diagnoses.

*‘[Smoking’s] maybe a contributing factor, but it’s not the entire cause. And I’ve just never thought along those lines. I thought, well, it [cancer diagnosis] was maybe meant to happen and that was that.’ (P26-Colorectal-Smoker)*

*‘Even yet I maintain that it’s not the cigarettes [that caused cancer].’ (P16-Head&Neck-Smoker)*

*‘I had cancer in the vagina, so it’s hardly related to my cigarettes…and because it wasn’t related to the cancer, my smoking, that's definitely why I didn't stop. If she [health professional] had said to me [that it was related to smoking] I’d have been off them […]’ (P17-Gynae-Smoker)*

While some patients did not acknowledge the causal relationship between smoking and their cancer, others thought that in the face of terminal illness or in light of their smoking history, that it was ‘too late’ and that there was little point in stopping. Indeed, when asked if they thought there would be any impact on their health if they stopped smoking, two participants told us,

*‘No. I don’t think so…I really think that I’m too far gone now…’ (P40-Lung-Smoker)*

*‘Well I honestly don't think it’ll do me any good, cause I'm 69, I've been smoking for 59 years. I think it’ll maybe do me more harm than good.’ (F6-Smoker)*

The three family members in our study cohort who had stopped smoking expressed that their decision to do so had been influenced, in varying degrees, by a relative’s cancer diagnosis and their subsequent perceptions about the links between smoking and health. In contrast, relatives’ cancer diagnoses, although clearly impacting emotionally and in other ways, were less influential as a motivator to stop smoking among family members who continued to smoke. Stronger influences that were mentioned included the perceived reduction of the social acceptability of smoking, caring responsibilities and cost.

*‘Well, I’m going to have to try [to quit] because if they keep putting the cigarettes up I’m not going to be able to afford them, that’s the thing. (F12-Smoker-Also diagnosed with lung cancer)*

*“I'm a mum, I can't stop for three days, I've still got to carry on and do things. I've still got to cook dinner, I've still got to - sometimes when I'm trying to quit I also get very irritable” (F4 – – Smoker)*

The impact of a terminal diagnosis on attitudes towards stopping smoking was also seen in some health professional responses, which are reported further below.

### Experiences and perceptions of smoking cessation services

Post-diagnosis, three of the 14 patients and each of the three family members who had stopped smoking reported doing so with varying degrees of support from community-based pharmacy smoking cessation services. Types of support included one-to-one support with a community pharmacist or participation in smoking cessation groups. Patients and family members in this group emphasised particularly the quality and person-centred relevance of information provided by pharmacy smoking cessation services and the effective interpersonal skills of smoking cessation facilitators.

*‘I mean, the girl [smoking cessation adviser] I spoke to, she was brilliant. She was an ex-smoker, so she talked about her own experience and the different routes you could go and the different things they could give you, the patches or the gum or […] the lozenges. We talked about my smoking habits and what would be the best route in relation to that.’ (P44-Lung-Ex-smoker)*

*‘Well, it [attending smoking cessation services] gave me more incentive not to smoke because I had a feeling that I’d let the nurse down if I had smoked that week […] and the fact that she was an ex-smoker herself was very good because she knew exactly what I was going through.’ (P50-Head&Neck-Ex-smoker)*

*‘I found the pharmacist very good because she explained it all to me.’* (F10-Ex-smoker)

Among continuing smokers (15 patients and 11 family members) just under half of each group stated an explicit desire to stop smoking. Repeatedly, these patients and family members expressed the belief that cessation could only be achieved through willpower and they did not indicate any consideration about using smoking cessation services. However, based on their own experiences, one patient (an ex-smoker) and one family member (a continuing smoker) reflected on the ‘appropriateness’ of current cessation services for those affected by a cancer diagnosis, particularly the lack of privacy when services were delivered in pharmacies and the repetitiveness of the generic ‘stop smoking’ message which, perhaps, was not effective for those people already affected by a cancer diagnosis.

*‘[A pharmacy is not] great if there's not an adviser there, and you're having to tell somebody in a very public place you've got lung cancer. I didn't like that bit of it.’ (F13-Smoker)*

*‘No, I’ve seen various numbers up on the ward in the hospital to contact for [smoking cessation] advice and all the rest of it, but I've been in touch with those before and it's the same thing, they tell you the same thing which you already know anyway.’ (P31-Head&Neck-Smoker)*

An additional, negative observation made by a patient who had decided to stop smoking using willpower rather than attend a smoking cessation group was that ‘there were not enough hours in the day’ to attend this type of service delivery regularly due to the various demands on patients’ time following a cancer diagnosis, particularly during treatment periods.

### Lack of meaningful discussion about smoking within the oncology service

From patients’ and family members’ perspectives, oncology staff rarely provided timely information about, or direct referral to cessation services. Around half of patients and most relatives recalled little or no discussion with health professionals about smoking.

*‘No, I always thought it was very strange how nobody ever said that I should stop smoking. I was waiting on it, I was waiting on all of them saying “You should really stop smoking”, nobody ever said it to me.’ (P3 -Colorectal, ex-smoker)*

*“I'm sitting here thinking once you'd [to wife] got diagnosed with cancer the last time there was nobody mentioned smoking….. [doctor’s name] knows that she smokes. I don't think she approves, don't get me wrong, she doesn't approve, there's nobody approves of you smoking but nobody’s going to come along and change that. You are a smoker and that's it.” (F2 - smoker).*

Some patients had anticipated and would have been receptive to staff being more proactive in encouraging smoking cessation and service uptake, as long as this was done sensitively and by the right person.

*‘Even if the nurse just took that two minutes to say, “Well we can help you. Have you ever thought about giving up? We're not here to make you do it, but have you ever thought about it?”.’ (P48-Gynae-Ex-smoker)*

*‘Thinking about it, why the hell was something not done?. Well, obviously as I say, it’s up to the individual again, but why did somebody not talk to me about it [smoking cessation] or, you know, whatever? But no, definitely nobody ever mentioned a thing. But I think it may help if you had the right person doing it, I quite believe it could help right enough. Definitely could.’ (P22-Lung-Smoker)*

*“I think probably it would’ve depended how they'd done it. If it was somebody who I liked and had confidence in and they did it the nice way, the right way, I would’ve probably said ‘yes you're quite right, we need to talk about this but not now when I'm ready for it cause I need some help’. Had they done it the wrong way, I can be fairly volatile…. I would be just as likely… to turn around and say ‘look mate, I've got enough on my mind at the moment knowing I could be pushing up daisies in six months and frankly whether I have another fag or not is totally immaterial to the situation’, so it would’ve depended very much on the person”. (P10 – Lung-Smoker)*

Patients were sometimes uncertain about whether family members who smoked should be involved in discussions about smoking cessation as they did not want to add to family problems or felt it was their own decision, even if they wished that the family member did not smoke. Although family members had not expected healthcare staff to talk to them about smoking cessation, many were open to discussing how they could support patients to stop and also how they themselves could access support.

*‘Yes…I’d probably have been quite open if somebody had phoned me or sent me a letter and said, you know, in the light of your brother’s diagnosis, or whatever, would you want to consider at this time getting some support for your own smoking. Yes, I probably would.’ (F14-Smoker)*

*‘That’s something I would have jumped right on. Although I wanted to quit to support [partner] more than myself, at that particular moment, although I did know I wanted to quit for myself too, it was because my biggest fear at the time was he would start smoking again and I didn’t want him to … I wanted him to have the best chance’ (F4CS -smoker).*

However, others were more resistant to being engaged in discussions about smoking cessation. Indeed, two family members felt that a direct approach from staff was intrusive or risked exploiting people’s vulnerability.

*‘[It] maybe [is] a good idea but, at the same time, you're catching… you'd be catching people at a real low.’ (F6-Smoker)*

*‘I'm sorry but […] at the point when [wife] was diagnosed with cancer, if somebody said to me “Well you'll need to stop smoking now”, I would’ve went “Aye, well you go and take a hike, sorry, I'm not in the frame of mind for that one”.’ (*F2GS*-Smoker)*

Participants, both patients and family members, suggested that specific and directly-targeted hospital-based cessation services, integrated with cancer treatment and care, enabling patients and families to combine participation with routine hospital attendance would be more likely to encourage successful uptake. However, our interviews suggest that the barriers expressed by patients and family members were often reinforced by health professionals. The following section illustrates how meaningful discussions about smoking and smoking cessation were frequently absent.

### Health professionals’ experiences and views

Overall, health professionals appeared to be more uneasy about talking about smoking and smoking cessation than were patients and family members. These were primarily professionals who did not have smoking cessation as their core or primary role (non-specialists). Their concerns stemmed from their perceptions and beliefs about the emotive and sensitive nature of the topic and expectations about how patients might react. Lack of opportunity for discussions, perceptions of their responsibility for talking about smoking cessation and lack of awareness about cessation services were also influential. When they did talk about smoking, relatively few discussed the benefits of cessation for future health. Very few health professionals indicated that they ever discussed smoking or smoking cessation with family members of people with cancer.

### Smoking as a sensitive issue

Interviews with healthcare professionals indicated their perceptions of smoking as a particularly sensitive issue to broach around the time of a patient’s diagnosis. Predominantly, fears of implying, instilling or exacerbating patients’ feelings of guilt about smoking made staff hesitant about raising the issue. There was uneasiness that arousing feelings of guilt could further upset patients already dealing emotionally with news of their diagnosis.

*‘[Patients] often may be feeling bad about it anyway, because they feel that they’ve brought this diagnosis on themselves because of their smoking habits, and although I’m a non-smoker myself, and don’t advocate smoking in any shape or form, I wouldn’t feel that it’s my position then to start lecturing them about that.’ (S17-Specialist Nurse)*

*‘…you don't want to be seen like you're telling them off or, you know, it’s already bad enough that they have a potentially terminal illness.’ (S1- Specialist Nurse)*

*‘I think one of the issues you have to be quite careful with is people are already beating themselves up about what they may have done wrong and why they are being punished by getting cancer. You don’t want to compound any feelings of guilt or self-loathing by preaching at them about their previous ‘evil ways’.’ (S8-Medical Specialist)*

Staff also expressed concern that raising the issue of smoking, particularly in the early stages of a cancer care pathway, might imply judgement by healthcare professionals. Being viewed as non-judgemental was considered important for maintaining their professional role in patients’ eyes and, for some staff, there was anxiety about assuming what might be interpreted by patients as a ‘policing’ role in relation to smoking.

*‘[Smoking] is not something that at that point, at that very first meeting, that's of any relevance because we need to develop a rapport, they can't feel that we’re being in any way judgemental or focusing on smoking.’ (S5- Specialist Nurse)*

*‘I think there’s too much anxiety. I think there’s an element of guilt or feeling like a blame game, and I think to, sort of, address it too aggressively up front can be detrimental to the doctor/patient relationship.’ (S7-Medical Specialist)*

*‘I think it was [staff] felt that they wanted to have a positive relationship with the patient from the beginning of their treatment, and that the patient should be able to bring concerns to them, and that if patients felt that they were policing their smoking that they might not have that positive relationship with them.’ (S16- Therapy Radiographer)]*

Given the perceived sensitivity of smoking within the context of a patient’s cancer diagnosis, the majority of staff indicated the tendency to delay or avoid raising it. During patients’ clinic attendance or treatment pre-assessments, the question ‘Do you smoke?’ often had to be asked alongside other lifestyle questions during completion of routine hospital documentation; however, some staff acknowledged that this did not necessarily lead to the initiation of discussion of the topic in any detailed way, unless a patient indicated their openness to engaging further in discussion about it. There was repeated mention by staff that they took a lead from patients themselves in their approach to talking about smoking and it was perceived as less risky to the clinician-patient relationship if the patient brought it up as an issue themselves or if they sensed that the patient was open to discussing it.

*‘You know, I would ask if they smoke and if they said ‘Yes’, and, “Oh, have you ever thought of giving up?”. Often, you get a flavour for where the patient is and how the consultation’s going, but sometimes it’s appropriate and sometimes it’s not been appropriate. I think if the patient brings it up then it’s sort of open goal if you like. It would be silly not to bring it up. ’ (S9-Medical Specialist)*

*‘..if there seems to be that actually there’s a readiness there to engage with it, then you can have quite a good conversation about options for smoking cessation and the benefits and that type of thing.’ (S5- Specialist Nurse)*

*‘I mean, I think, [smoking’s] something that’s very much on the agenda for discussing with patients but you have to be guided by the patient’s reactions to you bringing up the subject as well because there will be some who will just not entertain the idea of even considering cutting down, or anything, and you will have other patients who are a bit more amenable to having discussions about it.’ (S21-Specialist Nurse).*

When talking about how they approached the issue of smoking, healthcare staff often drew a distinction between patients with curable disease and those with incurable disease. This was an area where sensitivity about smoking was particularly marked. Those healthcare staff who mentioned this issue offered some explanations about why they might not raise the issue of smoking with patients with incurable cancer around the time of diagnosis. Reasons included the assessment that smoking may be a stress relief or coping mechanism for patients and it may offer them personal enjoyment. Additionally, within a palliative context smoking cessation may be considered of lesser priority than others (e.g. pain relief).

*‘…when you’ve got a curable cancer where there is good evidence that continuing to smoke will impact negatively on cure rates then I really do bang on about it and encourage them to quit….if we’re offering treatment that is purely aimed at enhancing quality of life then you may be doing them a dis-service getting them to or persuading them to stop…I would ask them if they smoked and obviously document a record of their smoking habit. Would I bang on at them to stop? No.’ (S10-Medical Specialist)*

*‘If a patient..were palliative in nature then I probably wouldn’t pursue that, and maybe that’s wrong on my part but reasons for that being if it’s palliative nature and their lifespan isn’t long anyway and that’s something they get enjoyment out of then I wouldn’t feel that was my place to suggest that they stop doing that.’ (S13-Specialist Nurse)*

*‘But I think sometimes I have felt maybe slightly guilty bringing it up because I know that it is a stress relieving thing. And I think maybe in the grand scheme of things, in the bigger picture, actually in the context of metastatic cancer, is it that important to bring it up at that point?’ (S9-Medical Specialist)*

### Perceptions of responsibility for talking about smoking cessation

Interviews suggested a picture of diffused responsibility for tackling the issue of smoking. In a busy clinical environment, it was not necessarily perceived as part of healthcare professionals’ role and there was reluctance to ‘bombard’ patients/families with smoking cessation messages.

*’I think it’s better handled by other staff groups than myself, if I’m absolutely honest. Again, to put it into context, I’ve probably spent thirty or forty-five minutes with a patient, they’ve had enough of me, they’ve heard what I’ve got to say and to be honest I just want to move on with my clinic.’ (S8-Medical Specialist)*

While most staff at least asked if patients smoked as part of an initial assessment, very few followed this with advice or support for cessation. Indeed, some health professionals acknowledged their lack of awareness of smoking cessation services and referral methods, and others were unsure of who was actually tackling the issue.

*‘I’ve certainly seen doctors frequently – probably nurses as well – advising patients not to smoke but without actually giving them a strategy for doing so, without giving them a contact detail.’ (S7-Medical Specialist)*

*‘I’m…unaware of, you know, what other practitioners are doing and…what’s already been said… so, it’s kind of getting that sense that, perhaps, there’s very few people talking about it because everybody thinks everybody… There’s, like, an assumption being made that it’s being discussed by everybody but in actual fact it possibly isn’t so’ (S15 – Therapy Radiographer)*

Discussions with family members about smoking were rarely reported. Barriers included the absence of family members in clinics, a focus on the patient, limited contact time within oncology consultations and underlying concerns about causing family tensions. As the following quote illustrates, discussions with family members about health were seen as the individual’s choice rather than the clinician’s priority.

*‘…it’s not really something I've really thought about to be quite honest but[…]unless another relative accompanies a patient to an appointment or is present when we were doing a home visit[…]it would be quite otherwise difficult to engage with them ‘cause ultimately it’s up to their choice whether they want to come to us and seek our advice.’ (S3-GP)*

### Strategies used by health professionals to promote and support smoking cessation

A minority of staff, those who approached the issue of smoking with patients ‘head on’, reported ways to overcome the barriers explored above. A key strategy was providing clinical evidence on the adverse effects of smoking on treatment outcomes and informing patients about the benefits of stopping smoking on side-effects, treatment outcomes and reduction in likelihood of recurrence.

*‘If I were raising it [smoking cessation] at all I would raise it at the time I was explaining the treatment because if I’m talking about side-effects, as I have to[…] the side-effects are worse in people who smoke.’ (S8-Medical Specialist)*

*‘[I will say] your treatment will be much more unpleasant if you carry on smoking, that your chance of cure will be less if you carry on smoking […] you have probably a one in three change of developing a secondary primary, another cancer, at a later date and that’s going to be higher if you carry on smoking. I tend towards the stark. My style tends towards the stark.’* (S19-Medical Specialist)

*‘And maxillofacial, oral cancer, it’s generally the highest contributory cause, so they are told “Smoking has given you this disease in the first place”. And that’s what I say to them, you know, “We can cure you of this, but you have to give up smoking”’*. (S5- Specialist Nurse)

One staff member, a surgeon, recognising the potential sensitiveness of smoking cessation, saw this as an impediment to the patient moving on in the care pathway and attempted to ‘de-moralise’ the issue quickly.

*‘Some say “This is my entire fault doctor”. I don’t care whose fault it was, we are going to move on from this. This is not about blame, it’s not about preaching, it’s not about telling you you’re a bad person, it’s about what is best for you. So I try and keep it self-directed and pragmatic and try and remove any moral or value overly from it because I think it gets in the way.’ (S19-Medical Specialist)*

A nurse showed how she introduced the topic of smoking cessation as a ‘normal’ part of what is already a personal conversation.

*“With the cancer side we approach very personal issues with regards to [for example] sexual dysfunction, so talking about smoking, no I don't have an issue with that whatsoever and do you know what I think the more relaxed you are and incorporate it as a part of, I act like I talk to everybody about it, absolutely everybody, this is a normal part of what we do when we’re talking to patients” (S23 – Specialist Nurse).*

Another useful technique appeared to be one of informal, ‘positive reinforcement’ of behaviour change. A Specialist Nurse (S6) described how expressing delight at smoking cessation attempts – ‘That’s great’, ‘That’s fantastic’ – could, in her experience, provide encouragement and support to patients.

Smoking cessation staff perceived a lack of explicit commitment to the promotion of smoking cessation within oncology services, and emphasised that leadership from consultant oncologists and a multidisciplinary approach was needed to change the culture, so that support to stop smoking became an integral part of the care pathway.

*‘It needs to come from the lead clinical management networks right through, that this [support for smoking cessation] has to be done as part of holistic care for that patient, you know, and I find that very difficult in each speciality that they don't have that part of holistic care, you know, as part of an agenda.’ (S4-Smoking Cessation Adviser)*

A range of staff participants acknowledged that including smoking cessation discussions within the context of treatment plans was a more acceptable way of enhancing patients’ perceptions of smoking risks and cessation benefits. One of the main ways in which this was thought to be possible was to improve links with the smoking cessation service within the hospital and to embed smoking cessation professionals more explicitly into multidisciplinary cancer teams.

## Discussion

To our knowledge, this is the first study in the UK to explore multiple perspectives - patients, family members and healthcare professionals - of the barriers and facilitators to increasing uptake of smoking cessation services within the context of a cancer diagnosis. Previous studies have examined these perspectives in relation to pregnant and new mothers, finding that women’s, partners’ and health professionals’ views and experiences all influence and challenge smoking cessation at a time that might be considered an opportunity for positive behaviour change [44, 45]. Our findings suggest that in the context of cancer care, smoking cessation support is currently insufficiently integrated into the care pathway and does not fully meet the needs of people affected by cancer. The low uptake of current smoking cessation services among patients with cancer and their relatives can be explained by a combination of individual, organisational and system factors, which serve to limit opportunities for smoking cessation discussion and further support. Therefore, interventions to improve uptake of smoking cessation in this context must target systems as well as individuals.

Many of the findings of this study are consistent with previous research that has examined barriers and facilitators to smoking cessation and to accessing smoking cessation services in a wide range of population groups [45–48]. We believe these barriers need to be addressed in any intervention to promote uptake of smoking cessation services in people with cancer.

In our study, patients and family members perceived willpower or ‘doing it themselves’ as the best or most appropriate way to stop. They viewed both licensed stop smoking medications (such as Nicotine Replacement Therapy) and behavioural support from services as unlikely to work for them. This perception, leading to low levels of service uptake, exists at the population level [46, 47] and is not unique to cancer patients. However, our study found that the emotional burden of a cancer diagnosis and the time-consuming and demanding nature of treatment meant that patients, family members and health care professionals avoided talking about smoking and that many opportunities for smoking cessation support were missed [47, 49]. As with other patient groups, although participants appeared to expect some mention or offer of discussions about smoking, many healthcare professionals were fearful of damaging therapeutic relationships or felt that such discussions were inappropriate or likely to be ineffective, therefore took the line of least resistance. A predominant focus on smoking as a *cause* of cancer appeared to emphasise concerns about exacerbating potential guilt and blame and inhibit discussions about smoking cessation as a current and future-oriented means of taking control, improving treatment outcomes and longer term health.

In terms of accessing smoking cessation services, patients and staff had poor understanding and low expectations of what services could offer. There was limited evidence that patients’, family members’ and health care professionals’ made meaningful connections between cancer, smoking and health [46, 50]. This served to further reduce the likelihood that ‘teachable moments’ were used and access to smoking cessation services promoted. Furthermore, family members suggested that their loved one’s cancer was not a sufficient motivator to stop smoking and that other reasons, such as the cost of smoking, were needed. Our findings confirm the ambivalent relationships between social support and smoking behaviour. Some family members who continue to smoke distance themselves from the diagnosis by disputing the link between smoking and disease, and many believe strongly that smoking cessation is an individual choice and that a decision to quit has to be motivated by the ‘right reason’ and initiated at the ‘right time’, not necessarily as a reaction to life-threatening disease [51]. Conversely, and in agreement with Ochsner et al. [52], we also found evidence that the support of close relatives was an important factor in determining both intention and ability to quit smoking.

Health professionals’, patients’ and family members’ accounts of advice on smoking are also consistent with previous research among other population groups [44, 53, 54]. Provision of information and advice on smoking too often stopped after asking the questions about smoking status without progressing to providing advice to stop or, crucially, an active referral to cessation services. Some of this was attitudinal – staff did not view advice or referral as appropriate for particular groups of patients, for example, those receiving palliative treatment. Other aspects related to knowledge – while staff had a good understanding of the links between smoking and lung cancer, and respiratory conditions like Chronic Obstructive Pulmonary Disease (COPD), there was some evidence that they either did not know or did not convey links with some other cancers [55, 56]. Nor did they fully appreciate the clear association between smoking cessation treatment outcomes and improved long term health. There was also little or no mention of the understanding between second hand smoke exposure and cancer, which is relevant to the advice given to family members as well as patients [57, 58]. This is an important issue that can be prevented through better training and prioritisation of early and ongoing education on smoking in clinical curricula and in practice. While health professionals need to be sensitive to not blaming patients for a cancer diagnosis, they should not mislead or ignore possible links, thus inadvertently undermining the opportunities that cessation can provide, including secondary prevention. This means that education and training must support the development of a more sophisticated understanding of their own biases, the patient’s personal beliefs and a confidence and ability to engage in an ongoing process of supporting patients to give up smoking. Discussions about how smoking can make the side effects of treatment worse may provide a useful route for clinicians to broach the topic of smoking cessation but it is important that these go beyond using the initial emotional trigger of the diagnosis and the motivational context that the clinical setting provides [5].

Other aspects were clearly organisational, where advice and referral to smoking cessation was not prioritised and efforts were uncoordinated. We identified a philosophical and organisational distinction, and therefore separation, between the clinical world of oncology and the health promoting world of smoking cessation. Multiple studies have illustrated that the chances of successfully stopping double if licensed pharmacotherapies on prescription are accessed, and double again if this is combined with behavioural support of the type offered by smoking cessation services in the UK and elsewhere [59–61]. There is clearly an urgent need for greater prioritisation and coordination of smoking cessation advice and referral within cancer services. This requires action at a number of levels, including closer working between smoking cessation and cancer care professionals and implementation of national guidance. In the UK, the NICE (National Institute for Health and Care Excellence) guidance for acute services [62] is particularly relevant, and elsewhere, other smoking cessation guidance should be applied [63, 64]. Current developments that affect how patients and family members approach smoking cessation, including a rapid rise in the use in many countries of e-cigarettes as an aid to smoking cessation, also needs to be taken into account [65, 66].

There is an evident need for services, supported by policy-makers, to better convey to the public (including cancer patients and their family members) what can be offered and how this will work. This requires greater tailoring of media campaigns (which have been poorly resourced in a number of jurisdictions in recent years [67, 68]) as well as more effective action and advice from health professionals. Our study shows that where health professionals used direct but supportive messages and clear evidence to convey the importance of cessation for *individuals*, this appealed to patients’ sense of coherence about the role of smoking cessation on their health and therefore helped to motivate smoking cessation. A study of healthy women also found that providing a ‘coherent’ explanation of the link between smoking and cancer increased both perceptions of vulnerability to cervical cancer, and intentions to stop smoking [50]. Effective tailoring of stop smoking messages and providing support for cessation is clearly important for smokers with cancer, especially as patients often held the view that it was up to them to stop smoking. Raising awareness of the existence of the services and the potential for these to address individual motivations for cessation also matters. Tools and resources to support evidence-based messages and promote a more integrated, coherent and consistent approach to smoking cessation across the cancer pathway are a potential way forward.

Our study provides supporting insights that are consistent with those of US researchers who have conducted extensive research in this area [49]. A recent systematic review and meta-analysis concluded that smoking cessation interventions that are delivered in clinical settings are particularly effective, and recommended more research into the challenges of integrating smoking cessation interventions into oncology settings [69]. This is likely to require multi-disciplinary strategies that pay attention to emotional, behavioural and practical issues, and are personalised and proactive, as well as reactive [5].

There are some limitations to our study. Although our sample was large and diverse, the generalizability of our findings may be limited by the focus on one cancer centre. We also experienced a number of challenges associated with recruiting family members, including patients attending clinic appointments unaccompanied, patients being reluctant to distribute information sheets and family members not returning reply slips. To mitigate these problems, we attended NHS smoking cessation groups and used posters at a local cancer support centre and an advertisement in the local press to recruit five additional participants.

## Conclusions

Our study provides important insights into the specific barriers and facilitators experienced in a UK/Scottish context that may also be relevant to other countries. In particular it illustrates the need for health service system change as well as attention to education, training and support for health care professionals. It is clear that patients and family members are likely to find it acceptable for health care professionals to broach the subject of smoking in supportive and constructive ways and that individualised assessment and tailoring of smoking cessation advice is crucial. A clear message from this study is that efforts to integrate smoking cessation interventions more effectively into routine practice are warranted and could pay dividends for patients and family members.

## Abbreviations

MRC: Medical Research Council
NHS: National Health Service
NICE: National Institute for Health and Care Excellence
NVIVO: A qualitative data analysis package produced by QSR International http://www.qsrinternational.com/what-is-nvivo
REC: Research Ethics Committee
SIMD: Scottish Index of Multiple Deprivation
UK: United Kingdom
US: United States
